# A causal relationship between antioxidants, minerals and vitamins and metabolic syndrome traits: a Mendelian randomization study

**DOI:** 10.1186/s13098-023-01174-y

**Published:** 2023-10-10

**Authors:** Junxian Li, Fengju Song

**Affiliations:** 1 Department of Blood Transfusion, Key Laboratory of Cancer Prevention and Therapy in Tianjin, National Clinical Research Center for Cancer, Tianjin’s Clinical Research Center for Cancer, Tianjin Medical University Cancer Institute & Hospital, Tianjin, China; 2 Department of Epidemiology and Biostatistics, Key Laboratory of Molecular Cancer Epidemiology in Tianjin, National Clinical Research Center for Cancer, Tianjin’s Clinical Research Center for Cancer, Tianjin Medical University Cancer Institute & Hospital, Tianjin Medical University, Tianjin, China

**Keywords:** Metabolic syndrome, Antioxidant, Mineral, Vitamin, Mendelian randomization

## Abstract

**Background:**

The available evidence regarding the association of antioxidants, minerals, and vitamins with the risk of metabolic syndrome (MetS) traits is currently limited and inconsistent. Therefore, the purpose of this Mendelian randomization (MR) study was to investigate the potential causal relationship between genetically predicted antioxidants, minerals, and vitamins, and MetS.

**Methods:**

In this study, we utilized genetic variation as instrumental variable (IV) to capture exposure data related to commonly consumed dietary nutrients, including antioxidants (β-carotene, lycopene, and uric acid), minerals (copper, calcium, iron, magnesium, phosphorus, zinc, and selenium), and vitamins (folate, vitamin A, B6, B12, C, D, E, and K1). The outcomes of interest, namely MetS (n = 291,107), waist circumference (n = 462,166), hypertension (n = 463,010), fasting blood glucose (FBG) (n = 281,416), triglycerides (n = 441,016), and high-density lipoprotein cholesterol (HDL-C) (n = 403,943), were assessed using pooled data obtained from the most comprehensive genome-wide association study (GWAS) available. Finally, we applied the inverse variance weighting method as the result and conducted a sensitivity analysis for further validation.

**Results:**

Genetically predicted higher iron (OR = 1.070, 95% CI 1.037–1.105, *P* = 2.91E−05) and magnesium levels (OR = 1.130, 95% CI 1.058–1.208, *P* = 2.80E−04) were positively associated with increased risk of MetS. For each component of MetS, higher level of genetically predicted selenium (OR = 0.971, 95% CI 0.957–0.986, *P* = 1.09E−04) was negatively correlated with HDL-C levels, while vitamin K1 (OR = 1.023, 95% CI 1.012–1.033, *P* = 2.90E−05) was positively correlated with HDL-C levels. Moreover, genetically predicted vitamin D (OR = 0.985, 95% CI 0.978–0.992, *P* = 5.51E−5) had a protective effect on FBG levels. Genetically predicted iron level (OR = 1.043, 95% CI 1.022–1.064, *P* = 4.33E−05) had a risk effect on TG level.

**Conclusions:**

Our study provides evidence that genetically predicted some specific, but not all, antioxidants, minerals, and vitamins may be causally related to the development of MetS traits.

**Supplementary Information:**

The online version contains supplementary material available at 10.1186/s13098-023-01174-y.

## Introduction

Metabolic syndrome (MetS) is a state characterized by a cluster of metabolic abnormalities, including hypertension, hyperglycemia, dyslipidemia, and abdominal obesity, which collectively increase the risk of cardiovascular disease and type II diabetes [1]. With a prevalence ranging from 10 to 50% among adults worldwide [2], MetS poses a significant threat to global health, with alarming rates in specific populations, particularly among elderly individuals in the United States [3]. The escalating prevalence of MetS and its association with increased mortality have made it a substantial burden on public health care systems and national finances [4, 5].

One of the key hallmarks of MetS is insulin resistance, resulting from various lifestyle factors such as aging, obesity, sedentary behavior, smoking, and sleep apnea [6]. Insulin resistance leads to elevated levels of reactive nitrogen oxides (RONS) and pro-inflammatory cytokines, triggering the activation of c-Jun N-terminal kinases (JNK1), nuclear factor kappa-light-chain-enhancer of activated B cells (NF-κB), and mitogen-activated protein kinase (MAPK) [7]. Consequently, oxidative stress occurs when there is an imbalance between the excessive production of reactive oxygen species (ROS) and reactive nitrogen species (RNS) and the body's antioxidant defense system [8]. This oxidative stress damages cellular components, such as lipids, proteins, and DNA, contributing to the development of various metabolic disturbances [9].

The potential roles of antioxidants, minerals, and vitamins in maintaining cellular health, reducing oxidative stress, and modulating metabolic pathways have been subjects of extensive research. These essential micronutrients have garnered considerable attention due to their proposed protective effects against specific cancers and other chronic diseases. Clinical data analysis has revealed low serum levels of retinyl esters, vitamin C, and carotenoids in MetS patients [10]. Moreover, growing evidence suggests that high intakes of nutrients, such as vitamin C [11], vitamin D [12] and calcium [13], could inhibit oxidative stress processes. Vitro experiments have shown that certain antioxidant nutrients reverse the inflammatory response caused by oxidative stress [14]. However, results from clinical trials examining the effectiveness of antioxidants as disease preventive agents have been inconsistent, with some even showing potential harm [15, 16]. These discrepancies may be attributed to limitations in observational studies (such as residual confounding and reverse causality) and challenges in randomized control trials, including low treatment compliance, inadequate dosages, short trial durations, and insufficient statistical power.

To overcome the bias of previous studies, we improved the study design through Mendelian randomization (MR) analysis, using genetic variation as an instrumental variable (IV), to establish reliable causal inferences between genetically predicted antioxidant, vitamin and mineral exposure levels and risk for MetS traits. By leveraging genetic variants as proxies for antioxidant, mineral, and vitamin levels, we aimed to obtain more robust and unbiased estimates of the potential causal effects of these micronutrients on MetS traits, thus contributing to a better understanding of preventive strategies for MetS and related conditions.

## Materials and methods

### Study design

**Fig. 1:**
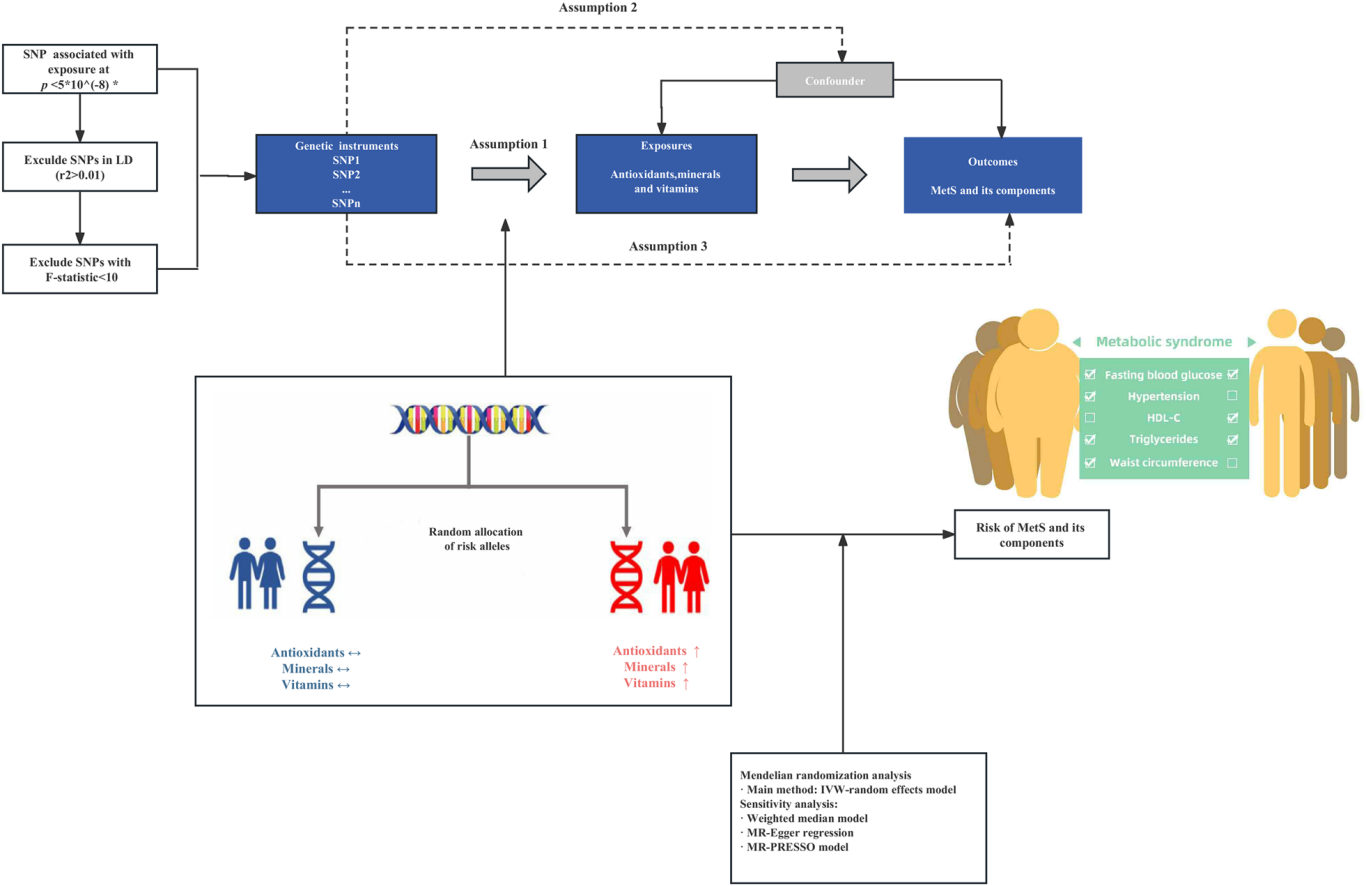
A flowchart of study design. Assumption 1 suggests that the genetic variants proposed as instrumental variables should be closely associated with the antioxidants, minerals and vitamins, SNPs should be associated with these circulating nutrients at the level of genome-wide significance (*P* < 5 × 10^–8^); for those traits detected by < 2 SNPs, include genome-wide associations significant for suggestive significance (*P* < 1 × 10^–5^) or validated SNPs, if available; Assumption 2 suggests that the genetic variants used should not be associated with potential confounders, and Assumption 3 suggests that the genetic variants selected should affect the risk of the outcome only through the risk factor and not through other pathways. MetS indicates metabolic syndrome; WC: waist circumference; HDL-C: high-density lipoprotein cholesterol; FBG: fasting blood glucose; TG: triglycerides; IVW: inverse-variance weighted

An overview of the study design and the assumptions of the MR study are shown in Fig. 1. MR is a genetic instrumental variable analysis that uses SNPs as IVs for risk factors of interest. SNPs are randomly assigned at meiosis and are therefore not subject to reverse causality bias. Firstly, we obtained available genetic variants for antioxidants, minerals and vitamins from a large-scale GWAS. Secondly, we selected pooled data from the GWAS meta-analysis for MetS and its components including waist circumference (WC), hypertension, fasting blood glucose (FBG), triglycerides (TG) and high-density lipoprotein cholesterol (HDL-C). Finally, a two-sample MR analysis and sensitivity analysis were performed to assess the causal relationship between exposure to dietary sources and the risk of MetS and its components. The MR depended on three key assumptions: (1) IVs are significantly associated with the exposure of interest; (2) IVs are not associated with any confounders of the exposure-outcome association; and (3) IVs affect outcome through exposure only [17].

Details of the data sources used in this study are summarized in Additional file 1: Table S1. To minimize racial mismatch, our analyzes were restricted to participants of European descent, all studies have been approved by the relevant institutional review boards, and informed consent have been obtained from the participants.

### Data sources for antioxidants, minerals, and vitamins and selection of instrumental variables

We searched PubMed and the GWAS catalogue for newly published GWAS studies on circulating levels of diet-related antioxidants, minerals and vitamins in populations of European descent. Taking into account the accessibility of the exposed GWAS studies and the need to reduce sample duplication with outcome data, 18 circulating nutrients were identified: antioxidants (lycopene [18], uric acid [19] and beta-carotene [20]), minerals (calcium [21], copper [22], iron [23], magnesium [24], phosphorus [25], selenium [26] and zinc [22]) and vitamins (vitamin A [27], vitamin K1 [28], vitamin E [29], vitamin D [30], vitamin C [31], vitamin B6 [32], vitamin B12 [33] and folate [33]).

For each exposure factor, eligible IVs were selected based on the three main assumptions of MR. Firstly, we included SNPs that met a genome-wide significance threshold (*P* < 5 × 10^–8^), and for those exposures detected by < 2 SNPs, suggestively significant genome-wide associations significant (*P* < 1 × 10^–5^) or validated SNPs were included if available. rs2108622 and rs11057830 were excluded due to F < 10. We displayed the SNPs associated with antioxidants, vitamins and minerals in Additional file 1: Table S2, and selected a total of 161 SNPs as IVs for the levels of three types of circulating nutrients.

### Data sources for MetS and its components

We used the most comprehensive GWAS summary-level data from UK Biobank [34], including 291,107 individuals (59,677 cases and 231,430 controls) with missing data for genotype, outcome and covariates. Individuals were defined as having MetS by meeting three or more of the following five criteria: blood pressure ≥ 130/85 mmHg or taking antihypertensive medication, serum glucose ≥ 6.1 mmol/L or taking glucose-lowering medication, serum triglycerides ≥ 1.7 mmol/L, WC > 102 cm in men and > 88 cm in women, HDL-C < 1.0 mmol/L in men and < 1.3 mmol/L in women. This GWAS data were adjusted for age, sex, 10 principal components and genotyping batches.

For waist circumference, we extracted GWAS summary data from the Medical Research Council Integrated Epidemiology Unit (MRC-IEU) [35], which included 462,166 subjects of European ancestry; for hypertension, summary statistics were also available from the MRC-IEU [35], which included 463,010 subjects; for FBG, pooled level data were obtained from the meta-analysis of glucose and insulin-related traits (MAGIC) [36], which included 281,416 individuals; for TG and HDL-C, summary-level statistics were extracted from the UK Biobank of over 400,000 participants [37]. To our knowledge, there was no sample overlap between exposure and outcome GWAS.

### Statistical analysis

The inverse variance weighting (IVW) method was used as the primary outcome of the MR analysis. For exposures with more than 3 SNPs, the estimates of variance were then combined using a random multiplicative effects inverse variance weighting method. For exposures detected by only 2 SNPs, a fixed-effects inverse variance weighting method was used. If the exposure had only 1 SNP, the Wald ratio method was performed, where the SNP-outcome association estimate was divided by its SNP-exposure association estimate to obtain a causal relationship.

In this study, Cochrane's Q test, the weighted median [38], Egger regression intercept [39] and MR-PRESSO global test [40] were used for sensitivity analysis to further examine heterogeneity and horizontal polymorphism. Cochrane's Q test was used to quantify heterogeneity across instrumental variables. A weighted median model could provide consistent estimates if at least 50 percent of the weights come from effective instrumental variables. MR-Egger intercept test was used to characterize the potential horizontal polymorphism. In addition, MR-PRESSO method could identify horizontal pleiotropic outliers for SNPs and provide the same results as IVW in the absence of outliers. Based on the above analysis, we used IVW as the main causal effect estimate and considered the consistency of all MR methods.

Statistical analyzes were performed using RStudio (version 4.2.1) and the R package "TwoSampleMR" and results were expressed as odds ratios (OR) and corresponding 95% CIs. Because of multiple comparisons, the significance level was corrected using the Bonferroni method. *P* value < 4.63 × 10^–4^ was considered a strong association, a *P* value between 4.63 × 10^–4^ and 0.05 was considered a potential association, and the *P*-values were two-sided.

## Results

### Study overview

**Fig. 2:**
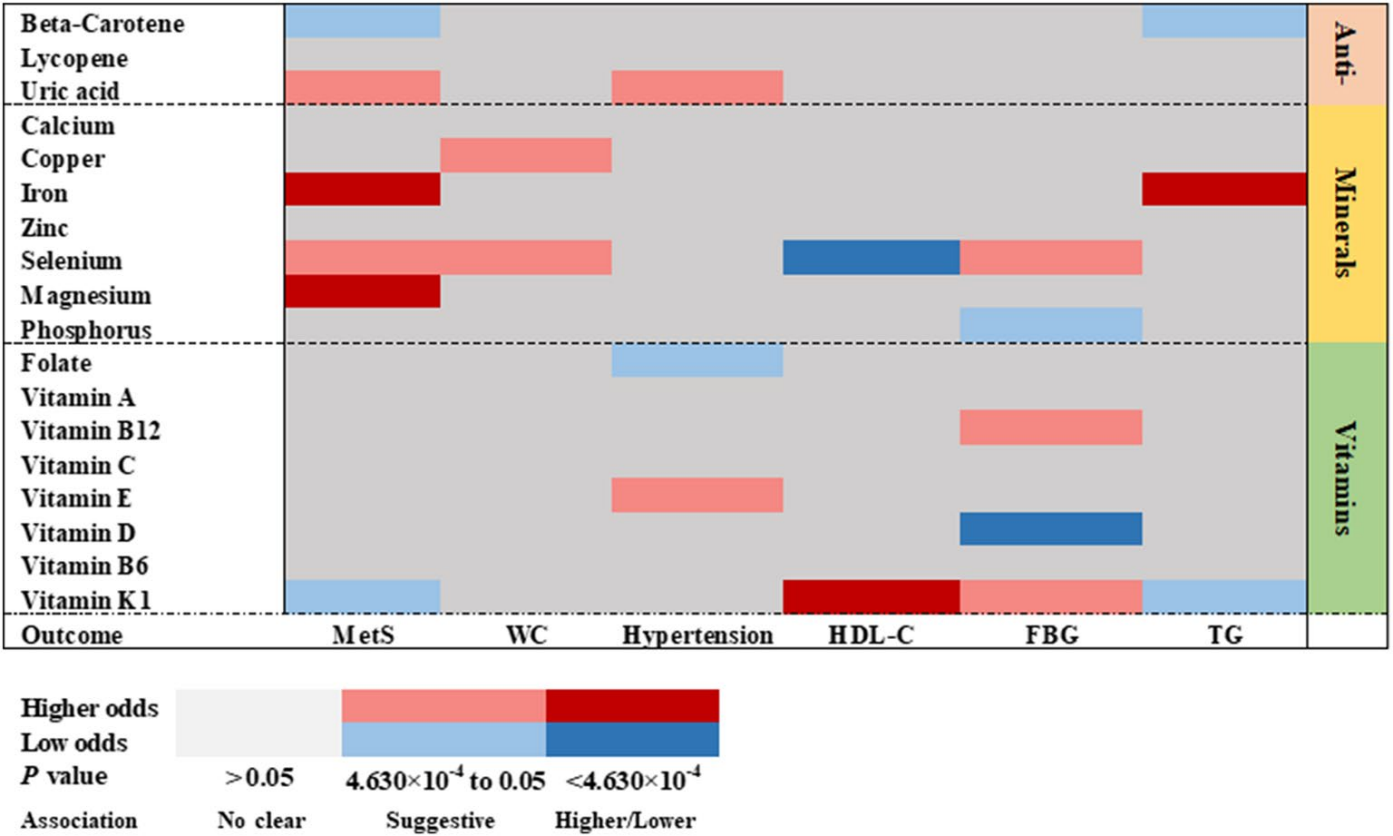
Primary analysis of associations between antioxidants, minerals and vitamins and MetS traits. MetS indicates metabolic syndrome; WC: waist circumference; HDL-C: high-density lipoprotein cholesterol; FBG: fasting blood glucose; TG: triglycerides

The current study assessed the causal impact of 18 genetically predicted antioxidants, minerals and vitamins in MetS traits such as WC, hypertension, HDL-C, FBG and TG. After a rigorous SNP filtering procedure, the number of SNPs finally used for each exposure varied from 1 to 81 (Additional file 1: Table S1). The F-statistic ranged from 11 to 10,000, suggesting that bias due to the use of weak instruments was unlikely (Additional file 1: Table S2). In the main analysis, 6 strongly causal and 15 potentially causal features were identified (Fig. 2).

### The causal role of antioxidants in MetS and its components

**Fig. 3:**
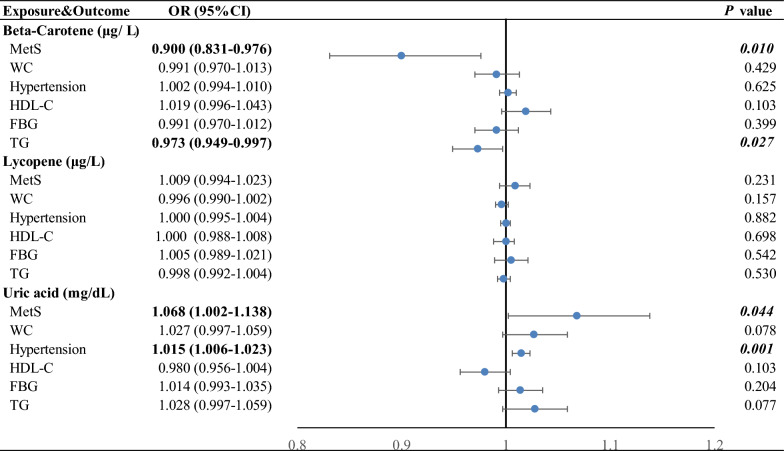
Associations of genetically predicted circulating antioxidants with risk of MetS and its components using the inverse-variance weighted mendelian randomization method. Estimated ORs represent the effect per unit increase in ln-transformed β-carotene, 1 μg/dL lycopene, and 1 mg/dL uric acid on MetS traits. The blue circles represent the OR and horizontal lines represent the 95% CI of the OR. *P* values below the Bonferroni-corrected threshold of 4.63 × 10^–4^ are displayed in bold and suggestive *P* values between 0.05 and 4.63 × 10^–4^ are displayed in bold-italic. OR indicates odds ratio; CI: confidence interval; MetS: metabolic syndrome; WC: waist circumference; HDL-C: high-density lipoprotein cholesterol; FBG: fasting blood glucose; TG: triglycerides

As to antioxidants, potential evidence was obtained for higher genetically predicted beta-carotene (OR per 1 SD increase in log-transformed ug/L, 0.900 [0.831–0.976]) with lower risk of MetS and higher genetically predicted uric acid levels with higher risks of MetS (OR per 1 mg/dL increase, 1.068 [1.002–1.138]) and hypertension (OR per 1 mg/dL increase, 1.015 [1.006–1.023]). A higher genetically predicted beta-carotene (OR per 1 SD increase in log-transformed ug/L, 0.973 [0.949–0.997]) was associated with lower odds of TG level (Fig. 3).

### The causal role of minerals in MetS and its components

**Fig. 4:**
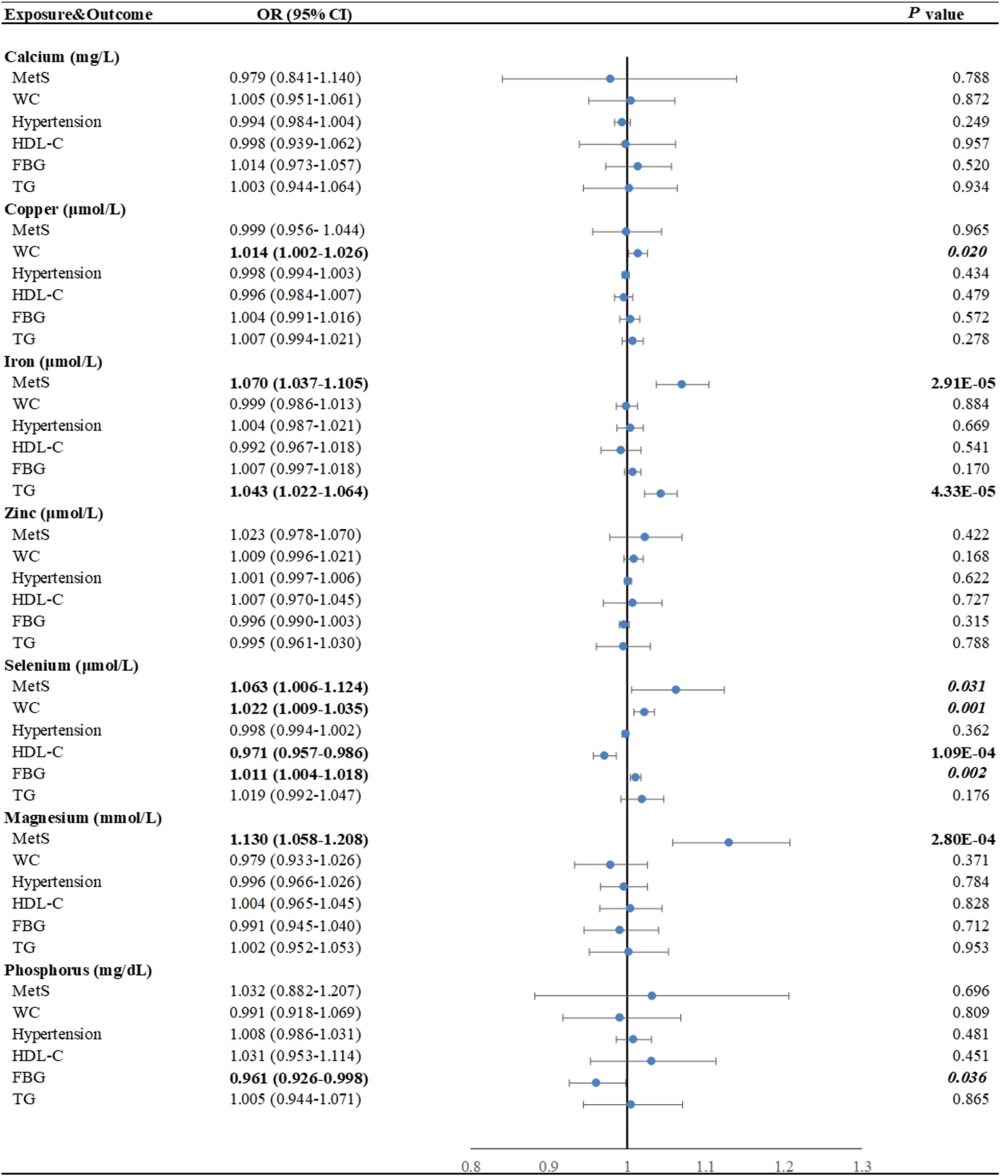
Associations of genetically predicted circulating minerals with risk of MetS and its components using the inverse-variance weighted Mendelian randomization method. Estimated ORs represent the effect per unit increase in ln-transformed copper, 1 sd iron, zinc, selenium, magnesium, phosphorus, and folate on MetS traits. The blue circles represent the OR and horizontal lines represent the 95% CI of the OR. *P* values below the Bonferroni-corrected threshold of 4.63 × 10^–4^ are displayed in bold and suggestive *P* values between 0.05 and 4.63 × 10^–4^ are displayed in bold-italic. OR indicates odds ratio; CI: confidence interval; MetS: metabolic syndrome; WC: waist circumference; HDL-C: high-density lipoprotein cholesterol; FBG: fasting blood glucose; TG: triglycerides

Among minerals levels, genetically predicted higher selenium levels were associated with a decreased risk of HDL-C, whereas genetically predicted iron and magnesium levels were positively associated with the risk of MetS (Fig. 4). The ORs per SD increase in genetically predicted circulating levels of these minerals were 0.971 (95% CI 0.957–0.986; *P* = 1.09E−04) for selenium in HDL-C, 1.070 (95% CI 1.037–1.105; *P* = 2.91E−05) for iron in MetS and 1.130 (95% CI 1.058–1.208; *P* = 2.80E−04) for magnesium in MetS. There was clear evidence that genetically determined iron was causally associated with TG (OR = 1.043, 95% CI 1.022–1.064, *P* = 4.33E−05).

We found a suggestive causal effect of copper (OR = 1.014, 95% CI 1.002–1.026, *P* = 0.020) and selenium (OR = 1.022, 95% CI 1.009–1.035, *P* = 0.001) on the risk of WC. Potential evidence showed that genetic liability to selenium was positively related to MetS (OR = 1.063, 95% CI 1.006–1.124, *P* = 0.031) and FBG (OR = 1.011, 95% CI 1.004–1.018, *P* = 0.002), while phosphorus negatively associated with FBG (OR = 0.961, 95% CI 0.926–0.998, *P* = 0.036).

### The causal role of vitamins in MetS and its components

**Fig. 5:**
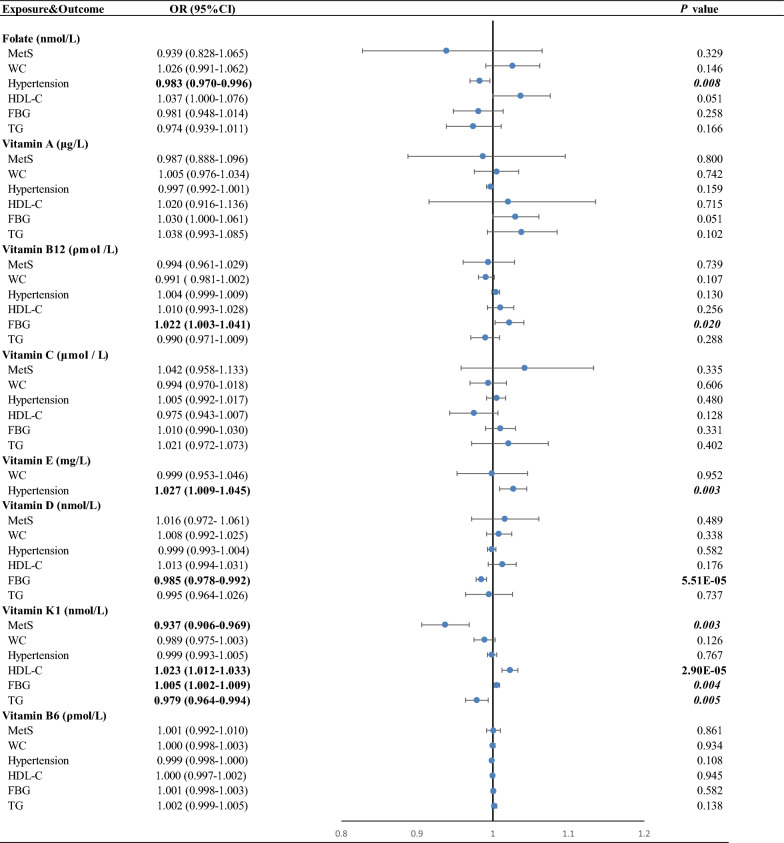
Associations of genetically predicted circulating vitamins with risk of MetS and its components using the inverse-variance weighted Mendelian randomization method. Estimated ORs represent the effect per unit increase in ln-transformed vitamin A and vitamin K1, 1 SD vitamin B6, vitamin B12, vitamin C, vitamin E and vitamin D on MetS traits. The blue circles represent the OR and horizontal lines represent the 95% CI of the OR. *P* values below the Bonferroni-corrected threshold of 4.63 × 10^–4^ are displayed in bold and suggestive *P* values between 0.05 and 4.63 × 10^–4^ are displayed in bold-italic. OR indicates odds ratio; CI: confidence interval; MetS: metabolic syndrome; WC: waist circumference; HDL-C: high-density lipoprotein cholesterol; FBG: fasting blood glucose; TG: triglycerides

Among vitamins levels, higher genetically predicted vitamin K1 levels were associated with an elevated risk of HDL-C levels (OR = 1.023, 95% CI 1.012–1.033, *P* = 2.90E-05), whereas genetically predicted vitamin D levels were negatively associated with risk of FBG levels (OR = 0.985, 95% CI 0.978–0.992, *P* = 5.51E−05) (Fig. 5).

There was suggestive evidence that genetically determined vitamin K1 level was causally associated with MetS (OR = 0.937, 95% CI 0.906–0.969, *P* = 0.003), FBG (OR = 1.005, 95% CI 1.002–1.009, *P* = 0.004) and TG (OR = 0.979, 95% CI 0.964–0.994, *P* = 0.005). Higher genetically predicted folate levels were associated with a decreased risk of hypertension (OR = 0.983, 95% CI 0.970–0.996, *P* = 0.008), whereas genetically predicted vitamin E levels were positively associated with the disease (OR = 1.027, 95% CI 1.009–1.045, *P* = 0.003). Genetically predicted vitamin B12 levels were positively associated with the risk of FBG (OR = 1.022, 95% CI 1.003–1.041, *P* = 0.020).

### Robustness of the results

To identify potential violations of the assumptions underlying MR, we performed sensitivity analyses employing three distinct methods: MR-Egger, weighted-median MR, and MR-PRESSO. Cochrane's Q test has not shown significant heterogeneity among the 6 strong causal associations. Weighted-median MR methods demonstrated consistent directions and similar effect estimates to IVW, except for the association between vitamin D and FBG levels. Furthermore, MR-Egger regression detected no evidence of directional pleiotropy for any outcome other than uric acid and WC or hypertension, and MR-Egger had pleiotropic *P* values > 0.05 after excluding outliers. As for potential associations, results from the sensitivity analyses of uric acid and vitamin K1 for MetS and its components were generally consistent with the primary analysis, though they did not always reach a significant level. The direction of the association did not alter after removing outliers in the MR-PRESSO analysis (Additional file 1: Tables S3–S7).

## Discussion

In this MR analysis, we have presented compelling evidence suggesting that genetically predicted higher levels of circulating iron and magnesium were associated with an increased risk of MetS. Regarding individual MetS components, we found an inverse relationship between genetically predicted selenium levels and HDL-C levels. Additionally, genetically predicted vitamin D levels were inversely associated with FBG levels. Conversely, we observed positive associations between genetically predicted vitamin K1 levels and HDL-C levels, as well as between iron levels and TG levels. Suggestive evidence supported associations between genetically predicted beta-carotene, uric acid, selenium, circulating folate, vitamin B12, vitamin E and vitamin K1 and MetS traits.

The potential link between beta-carotene and a reduced risk of MetS has garnered support from previous observational studies [41]. A cross-sectional study involving adolescents aged 12–19 years consistently found lower beta-carotene concentrations in the group with MetS compared to the group without the syndrome [42]. The underlying mechanisms proposed to explain this association include the direct impact of β-carotene on adipocyte function through its intracellular metabolites, retinaldehyde, and all-trans retinoic acid, both acting as ligands for nuclear receptors, which in turn repress adipogenesis [43]. Furthermore, compelling evidence suggested a correlation between genetic susceptibility to uric acid and an increased MetS risk, a finding consistent with prior prospective studies [44]and meta-analyses [45]. Yuan et al.'s meta-analysis, encompassing diverse populations, revealed a 30% rise in MetS risk for every 1 mg/dL increase in uric acid levels, demonstrating a notable linear dose–response relationship [45]. Additionally, Liu et al. unveiled a consistent and linear causal connection between heightened uric acid and the incidence of MetS, leading to the postulation of uric acid as a prospective individualized predictor for identifying systemic/hepatic metabolic abnormalities [46]. Therefore, lowering uric acid levels may be a potential therapeutic approach to prevent complex metabolic disorders.

Data on the associations of vitamins with MetS traits are scarce. We observed that genetic predisposition to higher vitamin K1 levels were associated with a decreased risk of low HDL-C level. Evidence from a cross-sectional and longitudinal analysis of a ten-year follow-up cohort suggests that high menaquinone intake and high vitamin K levels were associated with a lower incidence of MetS [47]. These associations were primarily driven by triglycerides and waist circumference. A cross-sectional study examined the relationship between phylloquinone intake and MetS and found that high phylloquinone intake was associated with lower prevalence of MetS (odds ratio = 0.72; 95% CI 0.25–2.09), although the association did not reach statistical significance [48]. Pan and Jackson [48] also studied the components of MetS and found that high phylloquinone intake was associated with a lower risk of low HDL-C, hypertriglyceridemia, and hyperglycemia. Epidemiological data concerning the association between vitamin D and FBG levels have produced inconsistent results, with both positive [49] and null findings [50] reported. Our study provides evidence supporting a causal role for vitamin D in MetS traits. In contrast, our study provides compelling evidence supporting a causal role for vitamin D in MetS traits. This role may be attributed to the mechanism of action of vitamin D on various physiological parameters. These mechanisms include the improvement of arterial stiffness, reduction of renin–angiotensin–aldosterone system activity, modulation of parathyroid hormone levels, regulation of inflammatory cytokines, enhancement of lipoprotein lipase activity, and promotion of improved phospholipid metabolism and mitochondrial oxidation [51]. Researches on vitamin K1 levels and FBG levels has not yet reached a unified conclusion. A randomized, controlled, crossover study inhibiting serum undercarboxylated osteocalcin (ucOC) by consumption of green leafy vegetables had no negative effects on glycemic status [47]. Therefore, further exploratory basic science or clinical inquiries are warranted to comprehensively understand the mechanism by which vitamin K1 contributes to the elevation of FBG levels.

The role of folate in hypertension risk has been previously noted. In line with our MR study results, a prospective cohort study involving 93,803 younger women aged 27 to 44 years from the Nurses' Health Study II and 62,260 older women aged 43 to 70 years from the Nurses' Health Study I demonstrated that higher total folate intake was associated with a reduced risk of incident hypertension, especially among younger women [52]. Mechanistically, folate deficiency can lead to the blockage of insulin synthesis and secretion in cultured β cells due to increased reactive oxygen species production and pro-apoptotic changes [53]. Conversely, folic acid has been shown to improve beta cell function by reducing oxidative stress, which is believed to contribute to the reduced risk of cardiovascular disease and diabetes by lowering insulinemia [54]. As for vitamin E, evidence from prospective cohort based on 24-h dietary records revealed a reverse J-shaped association between dietary vitamin E intake and new-onset hypertension in general Chinese adults [55]. As our MR study was not specifically designed to detect nonlinear associations, we can only infer that excessive vitamin E intake may be a causal risk factor for hypertension. Furthermore, our study's novel finding of a statistically potentially positive association between genetically predicted circulating vitamin B12 levels and FBG warrants confirmation through further research.

The role of minerals on MetS traits has been explored in previous observational studies [56, 57]. Findings from a cross-sectional study with 5323 participants from four Chinese megacities indicated that a positive association between high dietary iron intake and metabolic abnormalities [56]. Additionally, animal experiments have suggested that elevated dietary iron intake may lead to insulin resistance and oxidative stress [57]. Laboratory studies have provided insights into a possible mechanism, indicating that free iron possesses strong pro-oxidative properties, leading to oxidative damage and apoptosis through Fenton chemistry, resulting in the generation of RONs [58]. Contrasting our results, dietary magnesium intake has been reported to show an inverse association with MetS risk in studies conducted on both US [59] and Arab adults [60]. However, another Italian cross-sectional study did not observe such a statistical association [61]. In a 32-participant double-blind, placebo-controlled randomized study, the supplementation of magnesium did not substantially alter cardiometabolic biomarkers [62]. We posit that the disparities in findings could be attributed to magnesium's interplay with other nutrients. Interestingly, utilizing a validated genetic instrument, we observed that a genetic predisposition to high circulating magnesium was associated with an increased risk of MetS, suggesting that disturbances in magnesium metabolism may play a role in the pathological process of MetS pathogenesis.

The epidemiological data on the association between selenium and HDL-C levels show inconsistency, with both positive and null results reported. Our study provides evidence supporting a causal role for selenium in MetS traits. Recently, a case–control study from China discovered a positive association between selenium and MetS [63]. A recent meta-analysis demonstrated a positive relationship between selenium exposure and diabetes in epidemiological and experimental studies [64]. The positive association between selenium and hyperglycemia aligns with previous evidence that high selenium may have a diabetogenic effect. Our findings are consistent with this, as we observed that higher genetically predicted selenium levels were associated with increased odds of hypertriglyceridemia and a lower risk of developing low HDL-C levels [65]. The underlying mechanism explaining this association lies in the impact of selenium levels on the expression and activity of selenoproteins [66]. Excessive selenium levels may lead to an upsurge in ROS production, attributed to the increase of inorganic selenium in plasma [67]. The subsequent increase in ROS can lead to oxidative stress and insulin resistance [68], both of which are potential etiology of MetS.

The present study has several strengths, one of the main ones being the MR design. The study design reduced residual confounding and reversed causality, and strengthened causal inferences in the observed exposure-MetS traits associations. Another strength is that we have implemented the most comprehensive exposure dataset and the broadest summary-level data on MetS and its components, with no or very limited overlap between exposure and outcome data, so the ability to investigate causality is high, the type I error rate is low, and the estimated magnitude of effect is more accurate. The existing literature on the efficacy of micronutrient supplementation as a treatment for metabolic disorders is currently limited, comprising only a handful of small-scale studies focusing on specific patient subgroups. This scarcity of evidence poses challenges in formulating precise recommendations regarding nutritional supplementation. The results of this study will complement the evidence from current observational studies supporting causal relationships between circulating micronutrients and the development of metabolic disorders, which will contribute to the field of research in the nutritional prevention of MetS.

However, our study also has limitations. Firstly, we still cannot eliminate potential pleiotropic effects that may be masked by a few instrumental variables, although MR-egger intercepts showed little horizontal pleiotropy. Secondly, heterogeneity for some exposures was indicated by Cochran's Q values in the MR analysis. We therefore performed the MR-PRESSO analysis and the results indicated the stability of the observed associations. Thirdly, we restricted our analysis to individuals of European ancestry, which reduced potential bias introduced by demographics but limited the generalizability of our findings to other populations.

In conclusion, the results provided by this MR study support a causal relationship between multiple antioxidants, vitamins, and minerals and the risk of MetS and its components. Strategies targeting these modifiable factors, avoiding excessive urid acid, iron, magnesium, and selenium intake and increasing beta-carotene, vitamin K1 intake, can prevent MetS and lipid abnormalities and reduce the corresponding disease burden, and increasing vitamin D intake can reduce FBG level. Vitamin K1 have the potential to be chemoprotection against the pathogenesis of MetS traits. Our research might contribute significantly to the development of evidence-based recommendations for nutritional supplementation strategies targeting metabolic disorders.

### Supplementary Information

**Additional file 1: Table S1.** Characteristics of used studies and consortia. **Table S2.** Single nucleotide polymorphisms (SNPs) associated with circulating antioxidants, minerals and vitamins. **Table S3.** Associations of antioxidants, minerals and vitamins with MetS in sensitivity analyses. **Table S4.** Associations of antioxidants, minerals and vitamins with WC in sensitivity analyses. **Table S5.** Associations of antioxidants, minerals and vitamins with hypertension in sensitivity analyses. **Table S6.** Associations of antioxidants, minerals and vitamins with HDL-C in sensitivity analyses. **Table S7.** Associations of antioxidants, minerals and vitamins with FBG in sensitivity analyses. **Table S8.** Associations of antioxidants, minerals and vitamins with TG in sensitivity analyses.

## Data Availability

Data were available on request.
