# Supplementary material for: A causal relationship between antioxidants, minerals and vitamins and metabolic syndrome traits: a Mendelian randomization study
Source: Diabetol Metab Syndr. 2023 Oct 10;15:194. doi: 10.1186/s13098-023-01174-y (PMC10563368; doi:10.1186/s13098-023-01174-y)
Supplement: Supplementary file 1 — Additional file 1: Table S1. Characteristics of used studies and consortia. Table S2. Single nucleotide polymorphisms (SNPs) associated with circulating antioxidants, minerals and vitamins. Table S3. Associations of antioxidants, minerals and vitamins with MetS in sensitivity analyses. Table S4. Associations of antioxidants, minerals and vitamins with WC in sensitivity analyses. Table S5. Associations of antioxidants, minerals and vitamins with hypertension in sensitivity analyses. Table S6. Associations of antioxidants, minerals and vitamins with HDL-C in sensitivity analyses. Table S7. Associations of antioxidants, minerals and vitamins with FBG in sensitivity analyses. Table S8. Associations of antioxidants, minerals and vitamins with TG in sensitivity analyses. [file 13098_2023_1174_MOESM1_ESM.docx]

**Table S1.** Characteristics of used studies and consortia

**Table S2.** Single nucleotide polymorphisms (SNPs) associated with circulating antioxidants, minerals and vitamins

| **SNP** | **Exposure** | **Effect allele** | **Other allele** | **EAF** | ***P*-value** | **SE** | **Beta** | **R^2^** | **F-statistic** |
| --- | --- | --- | --- | --- | --- | --- | --- | --- | --- |
| rs2232315 | Lycopene | A | G | 0.03 | 1.00E-06 | 1.50E-01 | 0.740 | 3.187% | 24 |
| rs341075 | Lycopene | A | G | 0.02 | 6.00E-07 | 1.70E-01 | -0.870 | 2.967% | 26 |
| rs4635297 | Lycopene | A | C | 0.08 | 6.00E-07 | 5.00E-02 | 0.260 | 0.995% | 27 |
| rs6108801 | Lycopene | C | T | 0.04 | 4.00E-07 | 9.00E-02 | -0.480 | 1.769% | 28 |
| rs7680948 | Lycopene | A | C | 0.20 | 5.00E-09 | 3.00E-02 | -0.190 | 1.155% | 40 |
| rs4646068 | Uric acid | T | C | 0.69 | 7.71E-09 | 4.00E-03 | 0.024 | 0.025% | 36 |
| rs139428292 | Uric acid | A | G | 0.03 | 4.91E-08 | 1.30E-02 | -0.073 | 0.028% | 32 |
| rs11204701 | Uric acid | A | T | 0.78 | 1.05E-14 | 5.00E-03 | -0.036 | 0.045% | 52 |
| rs2760215 | Uric acid | T | C | 0.50 | 5.81E-11 | 4.00E-03 | -0.025 | 0.031% | 39 |
| rs12037861 | Uric acid | A | T | 0.70 | 3.39E-08 | 4.00E-03 | 0.023 | 0.022% | 33 |
| rs72782806 | Uric acid | A | G | 0.26 | 8.12E-09 | 4.00E-03 | 0.025 | 0.024% | 39 |
| rs12472381 | Uric acid | A | G | 0.39 | 1.80E-08 | 4.00E-03 | 0.022 | 0.023% | 30 |
| rs12987661 | Uric acid | T | C | 0.87 | 1.44E-12 | 6.00E-03 | 0.041 | 0.039% | 47 |
| rs17050272 | Uric acid | A | G | 0.42 | 1.57E-15 | 4.00E-03 | 0.032 | 0.050% | 64 |
| rs11683692 | Uric acid | T | C | 0.94 | 1.32E-08 | 8.00E-03 | -0.048 | 0.024% | 36 |
| rs1234413 | Uric acid | T | C | 0.44 | 7.08E-09 | 4.00E-03 | -0.022 | 0.024% | 30 |
| rs9287911 | Uric acid | A | T | 0.25 | 1.13E-17 | 4.00E-03 | 0.038 | 0.054% | 90 |
| rs187355703 | Uric acid | C | G | 0.98 | 2.70E-11 | 1.30E-02 | -0.086 | 0.036% | 44 |
| rs1047891 | Uric acid | A | C | 0.31 | 2.09E-08 | 4.00E-03 | -0.024 | 0.025% | 36 |
| rs9288447 | Uric acid | T | C | 0.55 | 3.27E-09 | 4.00E-03 | -0.023 | 0.026% | 33 |
| rs2581817 | Uric acid | C | G | 0.42 | 4.87E-35 | 4.00E-03 | 0.048 | 0.112% | 144 |
| rs7640441 | Uric acid | A | C | 0.25 | 1.26E-09 | 5.00E-03 | -0.028 | 0.029% | 31 |
| rs11718633 | Uric acid | T | C | 0.20 | 7.05E-09 | 5.00E-03 | -0.028 | 0.025% | 31 |
| rs80120242 | Uric acid | A | T | 0.95 | 1.87E-09 | 1.00E-02 | -0.062 | 0.039% | 38 |
| rs62294340 | Uric acid | A | G | 0.36 | 5.00E-08 | 4.00E-03 | -0.022 | 0.022% | 30 |
| rs62286563 | Uric acid | T | G | 0.98 | 1.16E-12 | 1.50E-02 | -0.103 | 0.044% | 47 |
| rs73224492 | Uric acid | A | G | 0.88 | 9.00E-59 | 6.00E-03 | -0.094 | 0.193% | 245 |
| rs98270 | Uric acid | A | G | 0.36 | 4.21E-08 | 4.00E-03 | 0.022 | 0.022% | 30 |
| rs10857147 | Uric acid | A | T | 0.71 | 2.21E-08 | 4.00E-03 | 0.024 | 0.024% | 36 |
| rs1481012 | Uric acid | A | G | 0.89 | 1.00E-200 | 6.00E-03 | -0.249 | 1.224% | 1722 |
| rs1440411 | Uric acid | T | C | 0.57 | 1.08E-12 | 4.00E-03 | -0.028 | 0.038% | 49 |
| rs455213 | Uric acid | T | C | 0.54 | 6.05E-12 | 4.00E-03 | -0.027 | 0.036% | 46 |
| rs76004499 | Uric acid | C | G | 0.97 | 3.27E-08 | 1.30E-02 | -0.074 | 0.030% | 32 |
| rs12530084 | Uric acid | T | C | 0.22 | 9.55E-48 | 5.00E-03 | 0.066 | 0.149% | 174 |
| rs198851 | Uric acid | T | G | 0.14 | 5.80E-13 | 5.00E-03 | 0.039 | 0.037% | 61 |
| rs742493 | Uric acid | T | C | 0.88 | 9.79E-11 | 6.00E-03 | 0.039 | 0.032% | 42 |
| rs1574430 | Uric acid | A | C | 0.41 | 2.77E-14 | 4.00E-03 | 0.029 | 0.041% | 53 |
| rs10223666 | Uric acid | C | G | 0.70 | 6.62E-28 | 4.00E-03 | 0.046 | 0.088% | 132 |
| rs4897160 | Uric acid | A | G | 0.48 | 1.96E-14 | 4.00E-03 | 0.030 | 0.045% | 56 |
| rs13226650 | Uric acid | A | G | 0.81 | 1.35E-23 | 5.00E-03 | 0.049 | 0.074% | 96 |
| rs10480300 | Uric acid | T | C | 0.28 | 4.26E-12 | 4.00E-03 | 0.030 | 0.036% | 56 |
| rs34861762 | Uric acid | T | C | 0.42 | 3.50E-19 | 4.00E-03 | 0.034 | 0.056% | 72 |
| rs2466077 | Uric acid | T | G | 0.53 | 1.78E-08 | 4.00E-03 | -0.022 | 0.024% | 30 |
| rs2943539 | Uric acid | T | C | 0.48 | 6.42E-28 | 4.00E-03 | 0.041 | 0.084% | 105 |
| rs10956924 | Uric acid | T | C | 0.28 | 1.79E-08 | 4.00E-03 | -0.024 | 0.023% | 36 |
| rs10971420 | Uric acid | T | C | 0.69 | 4.14E-14 | 4.00E-03 | 0.031 | 0.041% | 60 |
| rs56106601 | Uric acid | A | C | 0.95 | 2.68E-11 | 9.00E-03 | 0.061 | 0.038% | 46 |
| rs9420446 | Uric acid | T | C | 0.14 | 1.13E-11 | 6.00E-03 | -0.038 | 0.034% | 40 |
| rs35198068 | Uric acid | T | C | 0.71 | 5.85E-09 | 4.00E-03 | 0.025 | 0.026% | 39 |
| rs35506085 | Uric acid | A | G | 0.19 | 1.50E-08 | 5.00E-03 | -0.029 | 0.026% | 34 |
| rs148185902 | Uric acid | A | G | 0.01 | 5.76E-08 | 2.30E-02 | 0.123 | 0.036% | 29 |
| rs3925584 | Uric acid | T | C | 0.55 | 1.66E-15 | 4.00E-03 | 0.030 | 0.045% | 56 |
| rs71456318 | Uric acid | A | C | 0.48 | 4.41E-92 | 4.00E-03 | 0.079 | 0.312% | 390 |
| rs2022051 | Uric acid | A | G | 0.79 | 5.13E-48 | 5.00E-03 | -0.070 | 0.161% | 196 |
| rs10896028 | Uric acid | A | T | 0.65 | 4.10E-33 | 4.00E-03 | -0.048 | 0.106% | 144 |
| rs7315236 | Uric acid | T | C | 0.36 | 1.91E-13 | 4.00E-03 | 0.029 | 0.039% | 53 |
| rs12313306 | Uric acid | T | C | 0.25 | 6.74E-65 | 4.00E-03 | -0.076 | 0.214% | 361 |
| rs1800574 | Uric acid | T | C | 0.03 | 2.84E-12 | 1.20E-02 | -0.081 | 0.039% | 46 |
| rs28530689 | Uric acid | A | C | 0.51 | 1.27E-16 | 4.00E-03 | 0.032 | 0.051% | 64 |
| rs12423664 | Uric acid | A | G | 0.15 | 1.75E-13 | 6.00E-03 | 0.042 | 0.045% | 49 |
| rs7986094 | Uric acid | A | C | 0.30 | 1.74E-08 | 4.00E-03 | -0.024 | 0.024% | 36 |
| rs626277 | Uric acid | A | C | 0.59 | 2.69E-11 | 4.00E-03 | 0.026 | 0.033% | 42 |
| rs861536 | Uric acid | A | G | 0.62 | 2.16E-09 | 4.00E-03 | 0.024 | 0.027% | 36 |
| rs1478604 | Uric acid | T | C | 0.71 | 4.49E-10 | 4.00E-03 | -0.026 | 0.028% | 42 |
| rs2929508 | Uric acid | A | T | 0.26 | 3.65E-09 | 5.00E-03 | -0.029 | 0.032% | 34 |
| rs8040109 | Uric acid | A | C | 0.71 | 5.85E-09 | 4.00E-03 | 0.025 | 0.026% | 39 |
| rs2472297 | Uric acid | T | C | 0.25 | 1.50E-08 | 5.00E-03 | -0.028 | 0.029% | 31 |
| rs57737646 | Uric acid | T | C | 0.03 | 5.40E-14 | 1.20E-02 | -0.094 | 0.043% | 61 |
| rs55781567 | Uric acid | C | G | 0.66 | 1.11E-08 | 4.00E-03 | 0.023 | 0.024% | 33 |
| rs4997081 | Uric acid | C | G | 0.20 | 4.18E-10 | 5.00E-03 | -0.030 | 0.028% | 36 |
| rs8050136 | Uric acid | A | C | 0.40 | 2.34E-10 | 4.00E-03 | 0.025 | 0.030% | 39 |
| rs4788815 | Uric acid | A | T | 0.36 | 7.44E-11 | 4.00E-03 | -0.026 | 0.031% | 42 |
| rs57652769 | Uric acid | T | C | 0.31 | 8.56E-18 | 4.00E-03 | -0.036 | 0.055% | 81 |
| rs9925837 | Uric acid | A | G | 0.85 | 5.85E-15 | 5.00E-03 | -0.042 | 0.046% | 71 |
| rs11644696 | Uric acid | A | G | 0.48 | 1.41E-08 | 4.00E-03 | 0.022 | 0.024% | 30 |
| rs2453580 | Uric acid | T | C | 0.60 | 7.01E-10 | 4.00E-03 | 0.025 | 0.030% | 39 |
| rs57070985 | Uric acid | A | G | 0.65 | 2.04E-12 | 4.00E-03 | 0.029 | 0.038% | 53 |
| rs4808762 | Uric acid | T | C | 0.72 | 1.36E-08 | 4.00E-03 | -0.024 | 0.023% | 36 |
| rs2868194 | Uric acid | T | C | 0.41 | 8.90E-12 | 4.00E-03 | -0.027 | 0.035% | 46 |
| rs10414501 | Uric acid | C | G | 0.96 | 1.56E-12 | 1.80E-02 | -0.125 | 0.129% | 48 |
| rs7267595 | Uric acid | A | C | 0.51 | 3.15E-09 | 4.00E-03 | 0.023 | 0.026% | 33 |
| rs6119510 | Uric acid | T | G | 0.60 | 3.20E-09 | 4.00E-03 | -0.023 | 0.025% | 33 |
| rs142773928 | Uric acid | A | G | 0.17 | 3.77E-09 | 5.00E-03 | 0.032 | 0.028% | 41 |
| rs1800961 | Uric acid | T | C | 0.03 | 1.63E-10 | 1.20E-02 | -0.076 | 0.038% | 40 |
| rs219781 | Uric acid | T | G | 0.25 | 1.56E-08 | 4.00E-03 | -0.025 | 0.023% | 39 |
| rs12485100 | Uric acid | T | G | 0.17 | 2.44E-10 | 5.00E-03 | -0.033 | 0.031% | 44 |
| rs6564851 | Beta-Carotene | G | T | 0.39 | 1.60E-24 | 1.50E-02 | 0.149 | 1.056% | 99 |
| rs7501331 | Beta-Carotene | T | C | 0.24 | 1.60E-05 | 2.00E-02 | -0.067 | 0.164% | 11 |
| rs12934922 | Beta-Carotene | T | A | 0.44 | 5.90E-10 | 2.00E-02 | 0.139 | 0.952% | 48 |
| rs10491003 | Calcium | T | C | 0.09 | 4.80E-09 | 1.00E-02 | 0.054 | 0.048% | 29 |
| rs1550532 | Calcium | C | G | 0.31 | 8.20E-11 | 6.00E-03 | 0.036 | 0.055% | 36 |
| rs1570669 | Calcium | G | A | 0.34 | 9.10E-12 | 6.00E-03 | 0.036 | 0.058% | 36 |
| rs1801725 | Calcium | T | G | 0.15 | 8.90E-86 | 8.00E-03 | 0.142 | 0.514% | 315 |
| rs7336933 | Calcium | G | A | 0.85 | 9.10E-10 | 8.00E-03 | 0.044 | 0.049% | 30 |
| rs7481584 | Calcium | G | A | 0.70 | 1.20E-10 | 6.00E-03 | 0.036 | 0.054% | 36 |
| rs780094 | Calcium | T | C | 0.42 | 1.30E-10 | 6.00E-03 | 0.034 | 0.056% | 32 |
| rs1175550 | Copper | G | A | 0.22 | 2.51E-10 | 3.20E-02 | 0.198 | 1.345% | 38 |
| rs2769264 | Copper | G | T | 0.16 | 1.49E-20 | 3.40E-02 | 0.313 | 2.633% | 85 |
| rs1799945 | Iron | G | C | 0.15 | 1.10E-81 | 1.00E-02 | 0.189 | 0.911% | 357 |
| rs1800562 | Iron | A | G | 0.07 | 2.90E-97 | 1.60E-02 | 0.328 | 1.401% | 420 |
| rs7385804 | Iron | A | C | 0.62 | 1.40E-18 | 7.00E-03 | 0.064 | 0.193% | 84 |
| rs8177240 | Iron | G | T | 0.35 | 6.60E-20 | 7.00E-03 | 0.066 | 0.198% | 89 |
| rs855791 | Iron | G | A | 0.55 | 4.30E-139 | 7.00E-03 | 0.181 | 1.622% | 669 |
| rs11144134 | Magnesium | C | T | 0.08 | 8.21E-15 | 1.00E-03 | 0.070 | 0.072% | 4900 |
| rs13146355 | Magnesium | A | G | 0.44 | 6.27E-13 | 1.00E-03 | 0.050 | 0.123% | 2500 |
| rs3925584 | Magnesium | T | C | 0.55 | 5.20E-16 | 1.00E-03 | 0.060 | 0.178% | 3600 |
| rs4072037 | Magnesium | T | C | 0.54 | 2.01E-36 | 1.00E-03 | 0.100 | 0.497% | 10000 |
| rs448378 | Magnesium | A | G | 0.53 | 1.25E-08 | 1.00E-03 | 0.040 | 0.080% | 1600 |
| rs7965584 | Magnesium | A | G | 0.71 | 1.05E-16 | 1.00E-03 | 0.070 | 0.202% | 4900 |
| rs1697421 | Phosphorus | A | G | 0.49 | 3.47E-16 | 5.00E-03 | 0.050 | 0.125% | 100 |
| rs17265703 | Phosphorus | A | G | 0.85 | 6.24E-08 | 6.00E-03 | 0.036 | 0.033% | 36 |
| rs2970818 | Phosphorus | A | T | 0.09 | 4.04E-08 | 8.00E-03 | 0.047 | 0.036% | 35 |
| rs9469578 | Phosphorus | C | T | 0.92 | 5.15E-10 | 9.00E-03 | 0.059 | 0.051% | 43 |
| rs947583 | Phosphorus | C | T | 0.29 | 2.19E-09 | 5.00E-03 | 0.035 | 0.050% | 49 |
| rs949644 | Selenium | A | G | 0.65 | 2.03E-16 | 2.04E-02 | 0.167 | 1.275% | 68 |
| rs478651 | Selenium | T | C | 0.49 | 3.53E-13 | 2.23E-02 | 0.162 | 1.318% | 53 |
| rs921943 | Selenium | T | C | 0.70 | 9.40E-28 | 2.25E-02 | 0.246 | 2.533% | 119 |
| rs3797535 | Selenium | T | C | 0.07 | 2.42E-09 | 3.57E-02 | 0.213 | 0.592% | 36 |
| rs10514151 | Selenium | T | C | 0.07 | 1.42E-08 | 3.69E-02 | 0.209 | 0.569% | 32 |
| rs1532423 | Zinc | A | G | 0.37 | 6.40E-12 | 2.60E-02 | 0.178 | 1.477% | 47 |
| rs4826508 | Zinc | T | C | 0.25 | 1.40E-12 | 3.00E-02 | 0.210 | 1.654% | 49 |
| rs2120019 | Zinc | C | T | 0.79 | 1.55E-18 | 3.30E-02 | -0.287 | 2.733% | 76 |
| rs10882272 | Vitamin A | C | T | 0.38 | 7.00E-15 | 1.49E-02 | -0.087 | 0.356% | 34 |
| rs1667255 | Vitamin A | C | A | 0.37 | 6.00E-14 | 1.53E-02 | 0.090 | 0.377% | 34 |
| rs2108622 | Vitamin K1 | T | C | 0.30 | 8.78E-07 | 3.00E-02 | 0.160 | 1.075% | 28 |
| rs2192574 | Vitamin K1 | C | T | 0.11 | 8.23E-07 | 6.00E-02 | 0.280 | 1.535% | 22 |
| rs4645543 | Vitamin K1 | T | C | 0.05 | 2.00E-07 | 8.00E-02 | -0.420 | 1.612% | 28 |
| rs6862909 | Vitamin K1 | T | A | 0.01 | 1.20E-07 | 2.20E-01 | -0.940 | 1.750% | 18 |
| rs964184 | Vitamin E | G | C | 0.28 | 8.00E-12 | 2.80E-02 | 0.110 | 0.491% | 15 |
| rs10741657 | Vitamin D | G | A | 0.60 | 2.05E-46 | 7.00E-03 | -0.090 | 0.389% | 165 |
| rs10745742 | Vitamin D | C | T | 0.60 | 1.88E-14 | 7.00E-03 | -0.050 | 0.120% | 51 |
| rs12785878 | Vitamin D | G | T | 0.25 | 3.80E-62 | 7.00E-03 | -0.110 | 0.454% | 247 |
| rs17216707 | Vitamin D | C | T | 0.21 | 8.14E-23 | 8.00E-03 | -0.080 | 0.212% | 100 |
| rs3755967 | Vitamin D | T | C | 0.28 | 1.00E-200 | 8.00E-03 | -0.270 | 2.939% | 1139 |
| rs8018720 | Vitamin D | C | G | 0.82 | 4.72E-09 | 9.00E-03 | -0.050 | 0.074% | 31 |
| rs10051765 | Vitamin C | C | T | 0.34 | 3.64E-09 | 7.00E-03 | 0.039 | 0.068% | 31 |
| rs10136000 | Vitamin C | A | G | 0.28 | 1.33E-08 | 7.00E-03 | 0.040 | 0.065% | 33 |
| rs117885456 | Vitamin C | A | G | 0.09 | 1.70E-11 | 1.20E-02 | 0.078 | 0.097% | 42 |
| rs13028225 | Vitamin C | T | C | 0.86 | 2.38E-30 | 9.00E-03 | 0.102 | 0.255% | 128 |
| rs2559850 | Vitamin C | A | G | 0.60 | 6.30E-20 | 6.00E-03 | 0.058 | 0.162% | 93 |
| rs33972313 | Vitamin C | C | T | 0.97 | 4.61E-90 | 1.80E-02 | 0.360 | 0.803% | 400 |
| rs56738967 | Vitamin C | C | G | 0.32 | 7.62E-10 | 7.00E-03 | 0.041 | 0.073% | 34 |
| rs6693447 | Vitamin C | T | G | 0.55 | 6.25E-10 | 6.00E-03 | 0.039 | 0.075% | 42 |
| rs7740812 | Vitamin C | G | A | 0.59 | 1.88E-09 | 6.00E-03 | 0.038 | 0.070% | 40 |
| rs9895661 | Vitamin C | T | C | 0.82 | 1.05E-14 | 8.00E-03 | 0.063 | 0.119% | 62 |
| rs4654748 | Vitamin B6 | T | C | 0.52 | 8.30E-18 | 2.80E-01 | 1.450 | 1.300% | 27 |
| rs1131603 | Vitamin B12 | C | T | 0.06 | 4.90E-49 | 1.70E-02 | 0.190 | 0.407% | 125 |
| rs1141321 | Vitamin B12 | C | T | 0.63 | 3.60E-26 | 7.00E-03 | 0.061 | 0.173% | 76 |
| rs117456053 | Vitamin B12 | G | A | 0.98 | 1.90E-09 | 2.60E-02 | 0.160 | 0.100% | 38 |
| rs12272669 | Vitamin B12 | A | G | 0.01 | 3.00E-09 | 7.00E-03 | 0.510 | 0.515% | 5308 |
| rs1801222 | Vitamin B12 | G | A | 0.59 | 3.30E-75 | 7.00E-03 | 0.110 | 0.585% | 247 |
| rs2270655 | Vitamin B12 | G | C | 0.94 | 2.20E-13 | 1.80E-02 | 0.066 | 0.049% | 13 |
| rs2336573 | Vitamin B12 | T | C | 0.03 | 8.40E-59 | 7.00E-03 | 0.320 | 0.596% | 2090 |
| rs34324219 | Vitamin B12 | C | A | 0.88 | 1.10E-111 | 7.00E-03 | 0.210 | 0.931% | 900 |
| rs34528912 | Vitamin B12 | T | C | 0.04 | 2.10E-15 | 2.10E-02 | 0.170 | 0.222% | 66 |
| rs3742801 | Vitamin B12 | T | C | 0.29 | 1.70E-13 | 9.00E-03 | 0.045 | 0.083% | 25 |
| rs41281112 | Vitamin B12 | C | T | 0.95 | 8.90E-35 | 2.00E-02 | 0.170 | 0.275% | 72 |
| rs56077122 | Vitamin B12 | A | C | 0.34 | 4.80E-21 | 9.00E-03 | 0.087 | 0.340% | 93 |
| rs602662 | Vitamin B12 | A | G | 0.60 | 2.40E-139 | 7.00E-03 | 0.160 | 1.229% | 522 |
| rs1801133 | Folate | G | A | 0.67 | 9.50E-53 | 8.00E-03 | 0.096 | 0.408% | 144 |
| rs652197 | Folate | C | T | 0.18 | 1.40E-12 | 1.10E-02 | 0.069 | 0.141% | 39 |

**Table S3.** Associations of antioxidants, minerals and vitamins with MetS in sensitivity analyses

**Table S4.** Associations of antioxidants, minerals and vitamins with WC in sensitivity analyses

**Table S5.** Associations of antioxidants, minerals and vitamins with hypertension in sensitivity analyses

**Table S6.** Associations of antioxidants, minerals and vitamins with HDL-C in sensitivity analyses

**Table S7.** Associations of antioxidants, minerals and vitamins with FBG in sensitivity analyses

**Table S8.** Associations of antioxidants, minerals and vitamins with TG in sensitivity analyses
